# Supplementary material for: Bifidobacterium response to lactulose ingestion in the gut relies on a solute-binding protein-dependent ABC transporter
Source: Commun Biol. 2021 May 10;4:541. doi: 10.1038/s42003-021-02072-7 (PMC8110962; doi:10.1038/s42003-021-02072-7)
Supplement: Supplementary file 2 — Supplementary Information [file 42003_2021_2072_MOESM2_ESM.pdf]

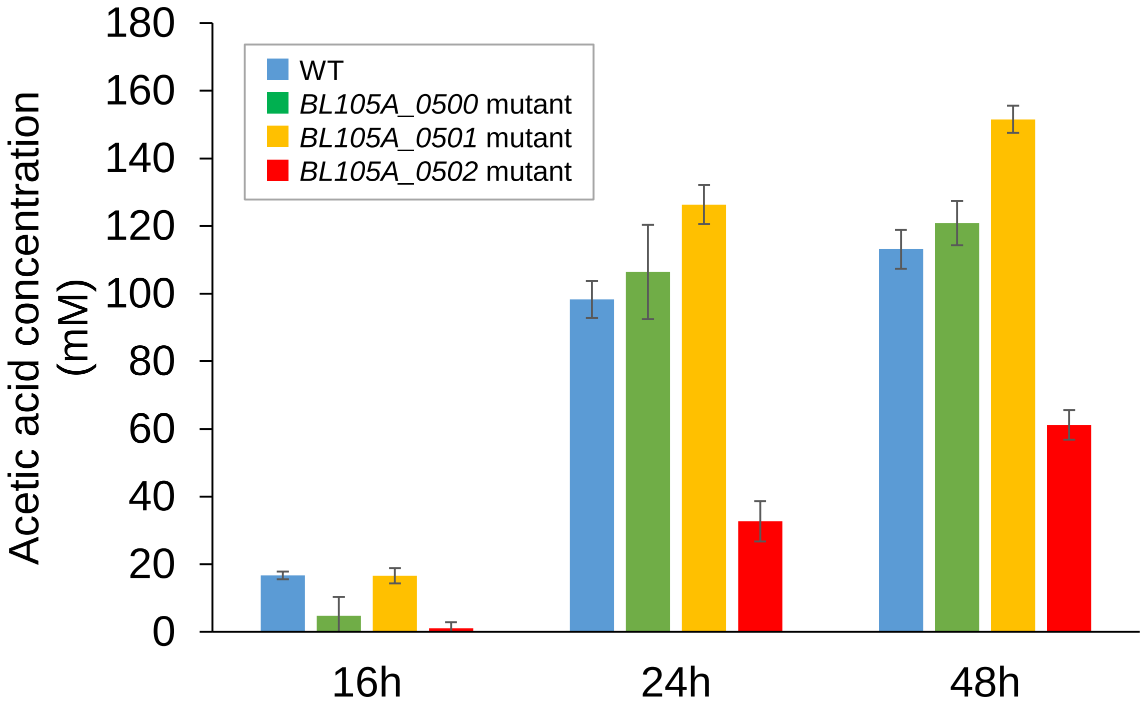

Supplementary Fig 1. Acetic acid concentration at the indicated time points in modified MRS medium supplemented with lactulose as the sole carbon source. The presented data are the mean ± SD of at least three independent assays.

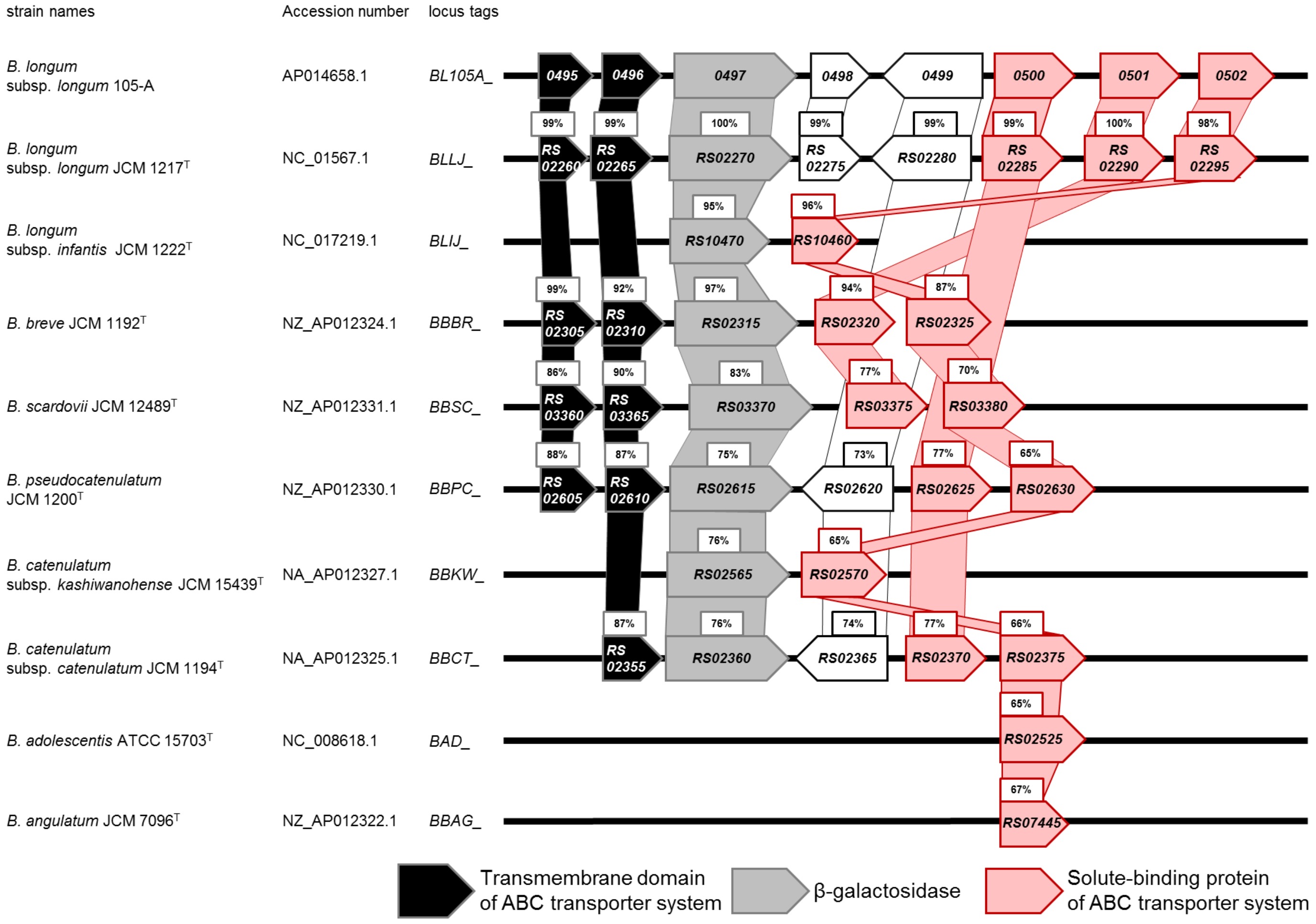

Supplementary Fig 2. Comparison of the genomic structure of lactulose-assimilating *Bifidobacterium* strains at the *BL105A_0502* locus. Solid arrows indicate open reading frames (ORFs) with their lengths proportional to the polypeptides chain lengths. The locus tags numbers are indicated inside the arrows. The amino acid identity (%) against homologue of *B. longum* subsp. *longum* 105-A is shown. The genes coding for transmembrane component, β-galactosidase, and SBP are coloured in black, grey, and light red, respectively.

Supplementary Table 1. Three *Bal* 6GBP orthologs of *B. longum* 105-A, identified by BLASTP analysis

Query	Subject	Identity (%)	Match length (bp)	E-value
Bal 6GBP	*BL105A_0500*	59.1	452	0
Bal 6GBP	*BL105A_0501*	73.7	448	0
Bal 6GBP	*BL105A_0502*	51.2	410	3.40E-138

Supplementary Table 2. Distribution of LT-SBP in *Bifidobacterium* species

Species	Number of strains	Number of strains with LT-SBP homologue
Bifidobacterium adolescentis	53	53
Bifidobacterium aemilianum	1	1
Bifidobacterium aerophilum	1	1
Bifidobacterium aesculapii	1	1
Bifidobacterium angulatum	7	7
Bifidobacterium anseris	1	1
Bifidobacterium apri	1	1
Bifidobacterium biavatii	2	2
Bifidobacterium bohemicum	3	3
Bifidobacterium bombi	2	2
Bifidobacterium boum	4	4
Bifidobacterium breve	108	108
Bifidobacterium callitrichidarum	1	1
Bifidobacterium callitrichos	5	5
Bifidobacterium castoris	1	1
Bifidobacterium catenulatum	16	16
Bifidobacterium choerinum	4	4
Bifidobacterium commune	1	1
Bifidobacterium criceti	1	1
Bifidobacterium dentium	21	21
Bifidobacterium dolichotidis	1	1
Bifidobacterium eulemuris	1	1
Bifidobacterium felsineum	1	1
Bifidobacterium gallinarum	3	3
Bifidobacterium goeldii	1	1
Bifidobacterium hapali	1	1
Bifidobacterium imperatoris	1	1
Bifidobacterium italicum	1	1
Bifidobacterium jacchi	1	1
Bifidobacterium lemurum	2	2
Bifidobacterium magnum	3	3
Bifidobacterium merycicum	5	5
Bifidobacterium minimum	3	3
Bifidobacterium mongoliense	3	3
Bifidobacterium moukalabense	13	13
Bifidobacterium myosotis	2	2
Bifidobacterium parmae	1	1
Bifidobacterium pseudolongum	75	75
Bifidobacterium pullorum	1	1
Bifidobacterium ramosum	2	2
Bifidobacterium reuteri	3	3
Bifidobacterium rousetti	1	1
Bifidobacterium ruminantium	3	3
Bifidobacterium saeculare	2	2
Bifidobacterium saguini	2	2
Bifidobacterium samirii	1	1
Bifidobacterium scaligerum	1	1
Bifidobacterium scardovii	5	5
Bifidobacterium stellenboschense	1	1

Bifidobacterium subtile	3	3
Bifidobacterium thermacidophilum	4	4
Bifidobacterium thermophilum	7	7
Bifidobacterium tibiigranuli	2	2
Bifidobacterium tsurumiense	4	4
Bifidobacterium longum	339	338
Bifidobacterium bifidum	95	94
Bifidobacterium pseudocatenulatum	76	68
Bifidobacterium actinocoloniiforme	3	0
Bifidobacterium animalis	81	0
Bifidobacterium aquikefiri	1	0
Bifidobacterium asteroides	14	0
Bifidobacterium avesanii	2	0
Bifidobacterium callimiconis	1	0
Bifidobacterium catulorum	1	0
Bifidobacterium coryneforme	3	0
Bifidobacterium crudilactis	1	0
Bifidobacterium cuniculi	2	0
Bifidobacterium gallicum	4	0
Bifidobacterium indicum	3	0
Bifidobacterium margollesii	1	0
Bifidobacterium primatium	1	0
Bifidobacterium psychraerophilum	3	0
Bifidobacterium simiarum	1	0
Bifidobacterium tissieri	3	0
Bifidobacterium vansinderenii	1	0
Bifidobacterium vespertilionis	2	0
Bifidobacterium xylocopae	1	0

Supplementary Table 3. Result of BLASTP analysis *BL105A_0502* against refseq protein

Query	Subject	Taxonomy	Identity (%)	Match length (bp)	Subject covarage	Query coverage
BL105A_0502	WP 173016859.1	*Lactobacillus rhamnosus*	98.595	427	100	100
	WP 116711921.1	*Gardnerella vaginalis*	65.105	427	100	100
	WP 081091204.1	*Gardnerella vaginalis*	64.871	427	100	100
	WP 082263263.1	*Gardnerella vaginalis*	64.637	427	100	100
	WP 102694610.1	*Gardnerella vaginalis*	63.934	427	100	100
	WP 004135396.1	*Gardnerella vaginalis*	63.934	427	100	100
	WP 019261216.1	*Gardnerella vaginalis*	63.934	427	100	100
	WP 116794172.1	*Gardnerella vaginalis*	63.7	427	100	100
	WP 103084803.1	*Gardnerella vaginalis*	63.466	427	100	100
	WP 004126765.1	*Gardnerella vaginalis*	63.466	427	100	100
	WP 041160604.1	*Gardnerella vaginalis*	63.232	427	100	100
	WP 075523540.1	*Gardnerella vaginalis*	63.232	427	100	100
	WP 064340159.1	*Gardnerella vaginalis*	62.998	427	100	100
	WP 004118866.1	*Gardnerella vaginalis*	62.998	427	100	100
	WP 165845980.1	*Gardnerella vaginalis*	62.998	427	100	100
	WP 174182833.1	*Gardnerella vaginalis*	62.998	427	100	100
	WP 033888514.1	*Gardnerella vaginalis*	62.998	427	100	100
	WP 102155703.1	*Peptoniphilus lacrimalis*	62.998	427	100	100
	WP 004107483.1	*Gardnerella vaginalis*	62.763	427	100	100
	WP 102703196.1	*Gardnerella vaginalis*	62.763	427	100	100
	WP 174182721.1	*Gardnerella vaginalis*	62.763	427	100	100
	WP 162863145.1	*Gardnerella vaginalis*	62.763	427	100	100
	WP 101896602.1	*Gardnerella vaginalis*	62.061	427	100	100
	WP 101889250.1	*Gardnerella vaginalis*	62.061	427	100	100
	WP 116689601.1	*Gardnerella vaginalis*	62.061	427	100	100
	WP 032817183.1	*Gardnerella vaginalis*	61.827	427	100	100
	WP 116285899.1	*Gardnerella vaginalis*	61.827	427	100	100
	WP 032813522.1	*Gardnerella vaginalis*	61.827	427	100	100
	WP 032815585.1	*Gardnerella vaginalis*	61.827	427	100	100
	WP 115793997.1	*Gardnerella vaginalis*	61.827	427	100	100
	WP 060786034.1	*Gardnerella vaginalis*	61.827	427	100	100

WP 032817960.1	*Gardnerella vaginalis*	61.827	427	100	100
WP 115761854.1	*Gardnerella vaginalis*	61.827	427	100	100
WP 004130652.1	*Gardnerella vaginalis*	61.827	427	100	100
WP 174191367.1	*Gardnerella vaginalis*	61.593	427	100	100
WP 032819329.1	*Gardnerella vaginalis*	61.593	427	100	100
WP 116692621.1	*Gardnerella vaginalis*	61.593	427	100	100
WP 004133268.1	*Gardnerella vaginalis*	61.593	427	100	100
WP 151215377.1	*Gardnerella vaginalis*	61.593	427	100	100
WP 060786792.1	*Gardnerella vaginalis*	61.358	427	100	100
WP 101891982.1	*Gardnerella vaginalis*	61.358	427	100	100
WP 101890332.1	*Gardnerella vaginalis*	61.358	427	100	100
WP 004138250.1	*Gardnerella vaginalis*	61.358	427	100	100
WP 119675767.1	*Gardnerella vaginalis*	61.358	427	100	100
WP 020759847.1	*Gardnerella vaginalis*	61.358	427	100	100
WP 116438730.1	*Gardnerella vaginalis*	61.358	427	100	100
WP 032812456.1	*Gardnerella vaginalis*	61.358	427	100	100
WP 004126136.1	*Gardnerella vaginalis*	61.124	427	100	100
WP 004120843.1	*Gardnerella vaginalis*	61.124	427	100	100
WP 009994176.1	*Gardnerella vaginalis*	61.124	427	100	100
WP 075038910.1	*Gardnerella vaginalis*	61.124	427	100	100
WP 004117715.1	*Gardnerella vaginalis*	61.124	427	100	100
WP 116437821.1	*Gardnerella vaginalis*	61.124	427	100	100
WP 004113506.1	*Gardnerella vaginalis*	61.124	427	100	100
WP 004114936.1	*Gardnerella vaginalis*	61.124	427	100	100
WP 048730195.1	*Gardnerella vaginalis*	61.124	427	100	100
WP 065189468.1	*Gardnerella vaginalis*	61.124	427	100	100
WP 116437116.1	*Gardnerella vaginalis*	61.124	427	100	100
WP 101886687.1	*Gardnerella vaginalis*	61.124	427	100	100
WP 116773091.1	*Gardnerella vaginalis*	61.124	427	100	100
WP 174191142.1	*Gardnerella vaginalis*	61.124	427	100	100
WP 032836469.1	*Gardnerella vaginalis*	60.89	427	100	100
WP 032833919.1	*Gardnerella vaginalis*	60.89	427	100	100

Supplementary Table 4. LT-SBPs specific primer pairs

	Forward primer	Reverse primer
B. adolescentis subtype	GTGGGAGCCGAGCATGAA	CCGGGCGTGTAGAAATCG
B. angulatum subtype	ATGGAAACGGTCGTGCAA	CGWGTAGAAGCTGCCGAAGT
B. longum subtype	CKCCCATGCCCACCTATG	TTCTGCCCGCCGAAATAC
B. scardovii subtype	CCACGAAGATCAGGACGTG	TGCCAGAATTCGGTGAAGAT

Supplementary Table 5. Faecal abundance of LT-SBP in different age groups

Total copy number of LT-SBP before ingestion (copy number per g-faeces)		20 - 64 y/o			65 - y/o			Total		
		Male (n = 112)	Female (n = 155)	Total (n = 267）	Male (n = 40)	Female (n = 87)	total (n = 127）	Male (n = 153)	Female (n = 241)	Total (n = 394）
High group	10^9 ≦	33.9%	33.5%	33.7%	17.5%	14.9%	15.7%	29.6%	26.9%	27.9%
Moderate group	10^7 - 10^9	50.9%	47.7%	49.1%	52.5%	48.3%	49.6%	51.3%	47.9%	49.2%
Low group	< 10^7	15.2%	18.7%	17.2%	30.0%	36.8%	34.6%	19.1%	25.2%	22.8%

Supplementary Table 6. Prevalence of the LT-SBP homologue based on metagenome data of the two different cohorts

Cohort	Japanese cohort[27]			Danish chohort[28]		
SRA number	DRP003048			PRJEB2054		
	Male (n = 57)	Female (n = 40)	Total (n = 97）	Male (n = 15)	Female (n = 18)	Total (n = 31）
LT-SBP prevalence	93.0%	85.0%	89.7%	30.8%	33.3%	29.0%

Supplementary Table 7. Composition of modified MRS medium

Components	Final concentrarion in dH_2O	Purity	Purchase source
Trypticase peptone	10 ($g\ L^{-1}$)		Becton, Dickinson and Company, New Jersey, USA
Beef extract	10 ($g\ L^{-1}$)		Becton, Dickinson and Company, New Jersey, USA
Granulated yeast extract	5 ($g\ L^{-1}$)		Becton, Dickinson and Company, New Jersey, USA
Tween-80	1 ($g\ L^{-1}$)		Nacalai Tesque, Inc., Kyoto, Japan
K_2HPO_4	2 ($g\ L^{-1}$)	> 99.0%	KOKUSAN CHEMICAL Co.,Ltd., Tokyo, Japan
Sodium acetate	5 ($g\ L^{-1}$)	> 98.5%	FUJIFILM Wako Pure Chemical Corporation, Osaka, Japan
Diammonium hydrogen citrate	2 ($g\ L^{-1}$)	> 99.0%	FUJIFILM Wako Pure Chemical Corporation, Osaka, Japan
$MgSO_4.7H_2O$	0.2 ($g\ L^{-1}$)	> 99.5%	FUJIFILM Wako Pure Chemical Corporation, Osaka, Japan
$MnSO_4.5H_2O$	0.05 ($g\ L^{-1}$)	> 99.9%	FUJIFILM Wako Pure Chemical Corporation, Osaka, Japan
Cysteine HCl [a]	0.5 ($g\ L^{-1}$)	> 99.0%	Kanto Chemical Co., Inc., Tokyo, Japan

[a] Cystein HCl was sterilized by filtration (0.2 μm) and added after autoclaving.

Supplementary Table 8. Primer pairs used for gene disruption, complementation, and confirmation

Target	Forward primer [a]	Reverse primer [a]
**For gene disruption in *B. longum* subsp. *longum* 105-A**		
BL105A_0500	CCAGCTCAAGGGATCtcgaggcaggctccggtgct	CGGTACCCGGGGATCggtcttggtgtcgatgagat
BL105A_0501	CCAGCTCAAGGGATCaggctggctccggtgctccg	CGGTACCCGGGGATCggtcttggtgtcgagcaggc
BL105A_0502	CCAGCTCAAGGGATCcctatggactgcccatggat	CGGTACCCGGGGATCggcgttggcaggctgccca
**For genotypic analysis of gene disruptants**		
BL105A_0500	atgaagttcggcaagaagacaattg	gcgtcgtggcaggcgtattcc
BL105A_0501	atgaagttcaccgttgctaag	ccgtggttggcgtactcgat
BL105A_0502	atgggcgaggtcatccgccgttt	tccgacagtacccggttgaa
**For *BL105A_0502* complementation**		
Promoter of *BL105A_0500*	GGAAAACTGTCCATAgccagttgtggagacgttac	cgttctttccttccttgttatg
Promoterless *BL105A_0502*	AGGAAGGAAAGAACGatgcgagtgatcggacagcg	AGGGCCTCGTGCATAcgtagtatgcaggaatcggc

[a] Uppercase letters represent 15 mer extension for In-Fusion cloning.

Supplementary Table 9. Synthesized nucleotide sequence of chloramphenicol-resistance gene sandwithced by *dnaA* promoter and terminator

Name	Nucleotide sequence[a]
Chloramphenicol-resistance gene sandwithced by *dnaA* promoter and terminator	aagcttTCGACACGTTGCGAGACACGCCTCAGATCTCTTATAATCGGCGGAATACCAACCATTCACGGCCAA<br>AAGTTATCCACAAATTACATGTTTGTTATTCACAATCCAATTATTTCAACACGCGACACGGTGGATAACTT<br>GTCTAAACCAGAGTAAATATTATCTGTTGTACATATTTCGCCACAGCGACAGAAGGGGCCACCatgaacttca<br>acaagatcgacctggacaactggaagcgcaaggagatcttcaaccactacctgaaccagcagaccaccttctccatcaccaccgagatcgacatctcc<br>gtcctgtaccgcaacatcaagcaggagggctacaagttctacccggccttcatcttcctggtcacccgcgtcatcaactccaacaccgccttccgcaccgg<br>ctacaactccgacggcgagctgggctactgggacaagctggagccgctgtacaccatcttcgacggcgtctccaagaccttctccggcatctggacccc<br>ggtcaagaacgacttcaaggagttctacgacctgtacctgtccgacgtcgagaagtacaacggctccggcaagctgttcccgaagaccccgatcccgg<br>agaacgccttctccctgtccatcatcccgtggacctccttcaccggcttcaacctgaacatcaacaacaactccaactacctgctgccgatcatcaccgcc<br>ggcaagttcatcaacaagggcaactccatctacctgccgctgtccctgcaggtccaccactccgtctgcgacggctaccacgccggcctgttcatgaact<br>ccatccaggagctgtccgaccgcccgaacgactggctgctgtgaCACAACGCTTCTGAGAGACGAAAAACGGCACTTCTTGG<br>ACTGACTTTCCGGAAAGTGCCGTTTTTGGTTGCTCAGGCGGATTCTGGTGGATACGTCGAGGCAACGGA<br>TTCGAGACATCAGATTCCTCATGGCTCCCCTTTATGAGGGGAGaagctt

[a] Uppercase letters represent the sequence of *dnaA* promoter or terminator, while lowercase letters indicate that of chloramphenicol-resistance gene. Lowercase letters with underlines represent HindIII sites.
