# Supplementary material for: Bifidobacterium response to lactulose ingestion in the gut relies on a solute-binding protein-dependent ABC transporter
Source: Commun Biol. 2021 May 10;4:541. doi: 10.1038/s42003-021-02072-7 (PMC8110962; doi:10.1038/s42003-021-02072-7)
Supplement: Supplementary file 3 — Description of Additional Supplementary Files [file 42003_2021_2072_MOESM3_ESM.pdf]

Description of Additional Supplementary Files

File name: Supplementary Data 1

Description: All source data underlying the graphs and charts presented in the main figures.
