# Supplementary material for: Bifidobacterium response to lactulose ingestion in the gut relies on a solute-binding protein-dependent ABC transporter
Source: Commun Biol. 2021 May 10;4:541. doi: 10.1038/s42003-021-02072-7 (PMC8110962; doi:10.1038/s42003-021-02072-7)
Supplement: Supplementary file 5 — Reporting Summary [file 42003_2021_2072_MOESM5_ESM.pdf]

nature research

Corresponding author(s): Keisuke Yoshida

Last updated by author(s): Mar 16, 2021

Reporting Summary

Nature Research wishes to improve the reproducibility of the work that we publish. This form provides structure for consistency and transparency in reporting. For further information on Nature Research policies, see our Editorial Policies and the Editorial Policy Checklist.

Statistics

For all statistical analyses, confirm that the following items are present in the figure legend, table legend, main text, or Methods section.

n/a	Confirmed	
☐	☒	The exact sample size (*n*) for each experimental group/condition, given as a discrete number and unit of measurement
☐	☒	A statement on whether measurements were taken from distinct samples or whether the same sample was measured repeatedly
☐	☒	The statistical test(s) used AND whether they are one- or two-sided<br>*Only common tests should be described solely by name; describe more complex techniques in the Methods section.*
☐	☒	A description of all covariates tested
☐	☒	A description of any assumptions or corrections, such as tests of normality and adjustment for multiple comparisons
☐	☒	A full description of the statistical parameters including central tendency (e.g. means) or other basic estimates (e.g. regression coefficient) AND variation (e.g. standard deviation) or associated estimates of uncertainty (e.g. confidence intervals)
☐	☒	For null hypothesis testing, the test statistic (e.g. *F*, *t*, *r*) with confidence intervals, effect sizes, degrees of freedom and *P* value noted<br>*Give P values as exact values whenever suitable.*
☒	☐	For Bayesian analysis, information on the choice of priors and Markov chain Monte Carlo settings
☒	☐	For hierarchical and complex designs, identification of the appropriate level for tests and full reporting of outcomes
☒	☐	Estimates of effect sizes (e.g. Cohen's *d*, Pearson's *r*), indicating how they were calculated

Our web collection on statistics for biologists contains articles on many of the points above.

Software and code

Policy information about availability of computer code

Data collection	The data were collected using Basic Local Alignment Tool (BLAST 2.6.0+), MUSCLE alignment tool (MUSCLE v3.8.31), Gblocks version 0.91b, PhyML 3.3, Phylip package v3.69, multiple sequence alignment using fast Fourier transform (MAFFT).
Data analysis	The data were analyzed using R software version 3.6.0, multcomp package in R.

For manuscripts utilizing custom algorithms or software that are central to the research but not yet described in published literature, software must be made available to editors and reviewers. We strongly encourage code deposition in a community repository (e.g. GitHub). See the Nature Research guidelines for submitting code & software for further information.

Data

Policy information about availability of data

All manuscripts must include a data availability statement. This statement should provide the following information, where applicable:

- Accession codes, unique identifiers, or web links for publicly available datasets
- A list of figures that have associated raw data
- A description of any restrictions on data availability

All datasets generated during and/or analyzed during the current study are available from the corresponding authors upon reasonable request.

Field-specific reporting

Please select the one below that is the best fit for your research. If you are not sure, read the appropriate sections before making your selection.

☒ Life sciences ☐ Behavioural & social sciences ☐ Ecological, evolutionary & environmental sciences

For a reference copy of the document with all sections, see nature.com/documents/nr-reporting-summary-flat.pdf

Life sciences study design

All studies must disclose on these points even when the disclosure is negative.

Sample size	The criteria for determining number of participants is based on the effect from previous experiment.<br>Sakai Y, Seki N, Hamano H, et al. A study of the prebiotic effect of lactulose at low dosages in healthy Japanese women. Biosci Microbiota Food Health. 2019;38(2):69-72. doi:10.12938/bmfh.18-013
Data exclusions	No data was excluded from analysis.
Replication	All growth profileing and real time PCR were repeated multiple independent times.
Randomization	Participants were randomly assigned to group A (lactulose first) or group B (placebo first). An assignment manager, who was independent of the trial staff, created the allocation order using the replacement block method. The allocation ratio was 1:1. According to the allocation order, the test food number was displayed on the test food package for each participant.
Blinding	The correspondence chart between the test food number and the assignment group was hidden from the authors, participants and trial staff, including the intestinal microbiome analyst and the statistician, until the completion of the trial. The trial staff assigned the test foods in ascending order of test food number, corresponding to the order of participant registration.

Reporting for specific materials, systems and methods

We require information from authors about some types of materials, experimental systems and methods used in many studies. Here, indicate whether each material, system or method listed is relevant to your study. If you are not sure if a list item applies to your research, read the appropriate section before selecting a response.

Materials & experimental systems

n/a	Involved in the study	
☒	☐	Antibodies
☒	☐	Eukaryotic cell lines
☒	☐	Palaeontology and archaeology
☒	☐	Animals and other organisms
☐	☒	Human research participants
☐	☒	Clinical data
☒	☐	Dual use research of concern

Methods

n/a	Involved in the study	
☒	☐	ChIP-seq
☒	☐	Flow cytometry
☒	☐	MRI-based neuroimaging

Human research participants

Policy information about studies involving human research participants

Population characteristics	Clininical stydy:<br>The inclusuion criteria were (1) age 18-31 years; and (2) defaecation frewuency 2-4 times/week.<br>The exclusion criteria were: (1) severe hepatic, renal, cardiac, gastrointestinal, cerebrovascular, endocrine, metabolic or infectious disease; (2) history of gastrointestinal resection; (3) gastrointestinal dysfunction, such as irritable bowel syndrome or inflammatory bowel disease; (4) use of medicines or supplements that could influence defaecation frequency (e.g. antibiotics, probiotics, laxatives, anti-diarrhoeal drugs and fibre); (5) milk allergy; (6) lactose intolerance; (7) participation in another study; and (8) individuals who were judged inappropriate for the study by the investigator or a physician.<br>Observational study:<br>Participans were between 0 and 104 years old (157 men and 210 women). Of over 80 years subjects were directly recruited by the authors to confirm that they were community dwellers.
Recruitment	Participants of the clininical stydy were healthy Japanese women who were students, staff member and affiliates of Showa Women's University.<br>Subjects of the observational stydy were community-dwelling Japanese volunteers.
Ethics oversight	The clinical study is registered with the University Hospital Medical Information Network Clinical Trials Registry (No. UMIN000027305).

The observational study was approved by the ethics committee of Kensyou-kai Incorporated Medical Institution (Osaka, Japan).

Note that full information on the approval of the study protocol must also be provided in the manuscript.

Clinical data

Policy information about clinical studies

All manuscripts should comply with the ICMJE guidelines for publication of clinical research and a completed CONSORT checklist must be included with all submissions.

Clinical trial registration	The protocol for this study is registered with the 178 University Hospital Medical Information Network Clinical Trials Registry (No. UMIN000027305).
Study protocol	Protocol can be accessed at University hospital Medical Information Network (UMIN) Center. (No. UMIN000027305: https://upload.umin.ac.jp/cgi-open-bin/ctr/ctr_view.cgi?recptno=R000031289)
Data collection	Participants were recruited between April and May 2017. The clinucal study was conducted at Showa Women's University (Japan) between May and December 2017. The data collected by a diary written by the participants.
Outcomes	The primary outcome was defaecation frequency and the secondary outcomes were the number of days on which defaecation occurred (defaecation days), faecal consistency and faecal volume. Fecal microbiota, moisture content and pH were also measured.
